# Hand Dexterity Impairment in Patients with Cervical Myelopathy: A New Quantitative Assessment Using a Natural Prehension Movement

**DOI:** 10.1155/2018/5138234

**Published:** 2018-07-04

**Authors:** Masao Omori, Satoshi Shibuya, Tsuyoshi Nakajima, Takashi Endoh, Shinya Suzuki, Shun Irie, Ryohei Ariyasu, Satoshi Unenaka, Hideto Sano, Kazutaka Igarashi, Shoichi Ichimura, Yukari Ohki

**Affiliations:** ^1^Department of Orthopaedic Surgery, Kyorin University School of Medicine, 6-20-2 Shinkawa, Mitaka, Tokyo 181-8611, Japan; ^2^Department of Integrative Physiology, Kyorin University School of Medicine, 6-20-2 Shinkawa, Mitaka, Tokyo 181-8611, Japan; ^3^Faculty of Human Development and Education, Uekusa Gakuen University, 1639-3 Ogura-cho, Wakaba-ku, Chiba 264-0007, Japan; ^4^Department of Physical Therapy, School of Rehabilitation, Health Sciences University of Hokkaido, 1757 Kanazawa, Tobetsucho, Ishikari-gun, Hokkaido 061-0293, Japan; ^5^Department of Sports Education, School of Lifelong Sport, Hokusho University, 23 Bunkyodai, Ebetsu, Hokkaido 069-0833, Japan

## Abstract

Cervical myelopathy (CM) caused by spinal cord compression can lead to reduced hand dexterity. However, except for the 10 sec grip-and-release test, there is no objective assessment system for hand dexterity in patients with CM. Therefore, we evaluated the hand dexterity impairment of patients with CM objectively by asking them to perform a natural prehension movement. Twenty-three patients with CM and 30 age-matched controls were asked to reach for and grasp a small object with their right thumb and index finger and to subsequently lift and hold it. To examine the effects of tactile afferents from the fingers, objects with surface materials of differing textures (silk, suede, and sandpaper) were used. All patients also underwent the Japanese Orthopedic Association (JOA) test. Preoperative patients showed significantly greater grip aperture during reach-to-grasp movements and weaker grip force than controls only while attempting to lift the most slippery object (silk). Patients, immediately after surgery, (*n* = 15) tended to show improvements in the JOA score and in reaction time and movement time with respect to reaching movements. Multiple regression analysis demonstrated that some parameters of the prehension task could successfully predict subjective evaluations of dexterous hand movements based on JOA scores. These results suggest that quantitative assessments using prehension movements could be useful to objectively evaluate hand dexterity impairment in patients with CM.

## 1. Introduction

Cervical myelopathy (CM) is a condition caused by spinal cord compression associated with disc herniation, cervical spondylosis, and congenital stenosis [1]. CM generally causes sensory disturbances of the upper and lower extremities (e.g., numbness or pain), reduced hand dexterity, gait disturbance, and urinary dysfunction. In severe cases, surgical treatment is applied to relieve compression of the spinal cord (i.e., decompressive surgery). Hence, appropriate functional outcome measures are necessary to determine the disease severity, progression of the disorder, and effectiveness of surgical treatment.

Several subjective scales have been developed to assess clinical deficits in CM patients: the European Myelopathy Score [2], Nurick Score [3], Cooper Myelopathy Score [4], and the Japanese Orthopedic Association (JOA) score [5]. The JOA score has been widely used in Japan, and high degrees of inter- and intraobserver reliability have been confirmed [6]. Upper and lower extremity impairments caused by CM have also been evaluated objectively using several scales. For example, with respect to the lower extremity, the 30 m walking test [7], 10 sec step test [8], and triangle step test [9] have been developed. As a scale for the upper extremity, the 10 sec grip-and-release test is frequently used in Japan to evaluate hand function in myelopathy patients [10]. Recently, our group also proposed functional assessment of the proximal arm muscle in CM patients using target-reaching movements [11]. However, movements in the 10 sec grip-and-release test are quite different from the daily actions performed with the upper extremity. In fact, patients with CM have difficulty in manipulating relatively small objects (e.g., buttons or tableware) with their fingers during activities of daily living (i.e., reduced hand dexterity), in which fine motor control of the fingers via various sensory feedback mechanisms is necessary. Therefore, we assume that a natural prehension movement could be useful as an objective measure for characterizing hand dexterity impairments. To verify this hypothesis, we first compared preoperative patients and age-matched control subjects using a newly developed prehension task, which involved asking patients to reach and pick up objects of various surface textures [12]. We aimed to determine if this prehension task is suitable for assessing hand dexterity in patients with CM.

## 2. Materials and Methods

### 2.1. Participants

Twenty-three patients diagnosed with cervical spondylosis myelopathy (15 men and eight women; 65.0 ± 14.8 years old, mean ± SD) and 30 age-matched controls (12 men and 18 women; 63.4 ± 17.2 years old) participated in this study. A chi-squared test with Yates' correction confirmed that participant type (i.e., patients and controls) and gender were independent of each other (*χ* ^2^_1_ = 2.38, *p* > 0.12). Mean symptom duration was 18.0 ± 23.5 months. Neurological segment diagnosis indicated impairments of the C3 level (*n* = 1), C4 level (*n* = 1), C5 level (*n* = 8), C6 level (*n* = 8), and C7 level (*n* = 5). Magnetic resonance imaging was used in all patients both pre- and postoperatively. Signal changes were detected in all but six patients on T2-weighted imaging but in only seven patients on T1-weighted imaging. All participants were right-handed and had normal or corrected-to-normal vision. All were unaware of the purpose of the experiment. This study was approved by the institutional human review committee at the Kyorin University School of Medicine (approval number 498), and all participants provided written informed consent in accordance with the Helsinki Declaration. Fifteen of the 23 patients (65%) who participated in the preoperative experiment underwent decompression surgery using expansive open-door laminoplasty (*n* = 13) or anterior spinal fusion (*n* = 2). Immediately after surgery (14.6 ± 6.5 days), they underwent the assessment a second time.

### 2.2. JOA Score

As a conventional clinical scale based on patient symptoms, the JOA score for CM was applied to all participants (Table 1). Although the JOA score originally included the functions of the lower extremity and bladder, only the scores regarding the upper extremity were used for two domains in the present study: motor function of the fingers (AI in Table 1: range 0–4) and sensory function of the upper extremity (BI in Table 1: range 0–2). Accordingly, the total score of motor and sensory functions ranged from 0–6 (AI + BI).

### 2.3. Apparatus and Procedures

**Figure 1:**
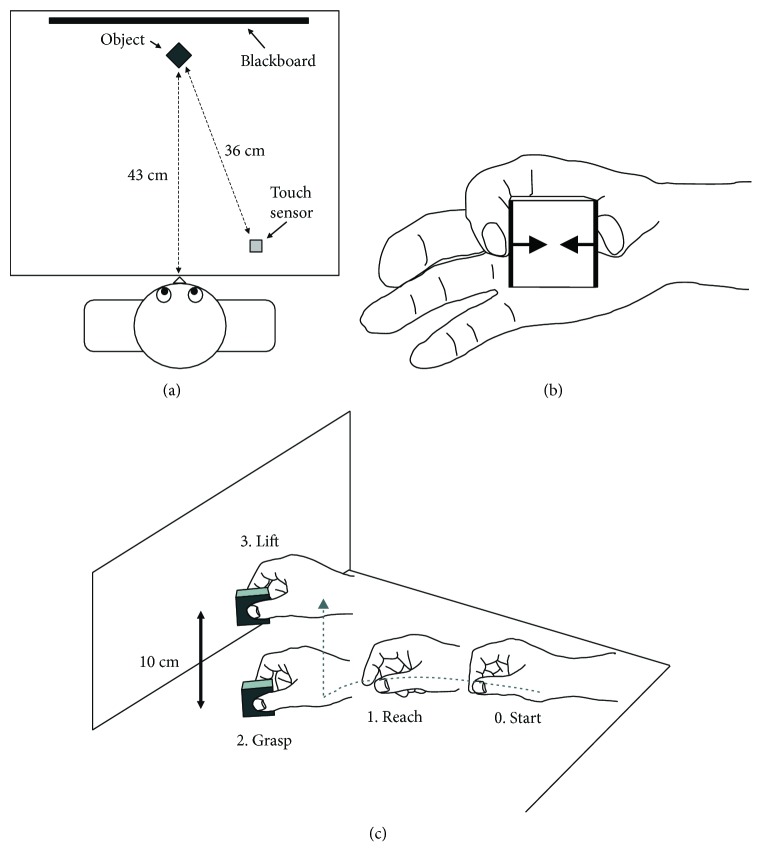
Experimental setup and prehension task. (a) Top view of the experimental setup. (b) Grip force during holding the object. The object had a force measurement system, which could measure perpendicular force to each side exerted to each side by the thumb or index finger. (c) Prehension task included three movement components: (1) reaching, (2) grasping, and (3) lifting.

Participants were seated in front of a wooden desk, on which a cubical object (4 cm per side, 70 g weight) was positioned in the participant's midsagittal plane, 43 cm from the front edge of the desktop (Figure 1(a)). A detachable surface material selected from silk, suede, or sandpaper was attached on two sides, which faced each other and were contacted by the participant's thumb and index finger (thick lines in Figure 1(b)). The object had an integrated force measurement system (see below), which could measure the perpendicular force exerted to each side by the thumb or index finger (i.e., grip force; arrows in Figure 1(b)). To detect movement initiation, an electrostatic touch sensor (2 × 2 cm) was positioned 13 cm to the participant's right side. The center-to-center distance between the object and the touch sensor was 36 cm. A blackboard stood immediately behind the object, on which a marker was attached 10 cm from the desktop as a height target when lifting the object.

All participants completed three conditions with different surface materials, the order of which was fixed to prevent slips: sandpaper, suede, and then silk (i.e., from the least slippery material to the most slippery). Each condition was composed of 10 successive trials. Before each trial, participants were instructed to position their right hand on the touch sensor and put tips of their right thumb and index finger together (“Start” in Figure 1(c)). After delivery of a first auditory stimulus (a pure tone at 500 Hz) as the “go” signal, participants reached for and grasped the object with their right thumb and index finger as soon and accurately as possible (“Reach and Grasp” in Figure 1(c)). Subsequently, they lifted the object to the target marker on the blackboard and held it there until a second tone was provided approximately 3 s after the initiation of holding (“Lift” in Figure 1(c)). After the second tone, the participant was allowed to put the object on the desktop again and return their right hand to the starting position. The participants took a rest of 3 min between conditions, during which the experimenter altered the surface material. In total, the experiment took about 20–30 min to complete.

### 2.4. Data Acquisition and Dependent Variables

Using an electromagnetic motion tracking system (The MotionMonitor; Innovative Sports Training, Inc., Chicago, IL, USA), the following three positions of the right hand were sampled three-dimensionally at 100 Hz: tips of the thumb, index finger, and wrist (i.e., styloid process of the radius). Grip force signals from the thumb and index finger were measured by two channels of strain gauges (KFG-2N-120-C1-11L1M2R, Kyowa Co., Tokyo, Japan) at 100 Hz that were mounted on the surfaces of two sides of the object. The grip forces from the two channels were averaged. Movement initiation was detected by the touch sensor.

Using the SC/ZOOM system (Physiology Section; IMB, University of Umea, Sweden), we extracted the following seven parameters from the raw data; (1) reaction time (RT): time from the auditory “go” signal to movement initiation; (2) movement time (MT): time from movement initiation to the time at which either the thumb or index finger contacted the object first (i.e., touch object); (3) maximum grip aperture (MGA): maximum distance between the thumb and index finger during the reach-to-grasp movement; (4) time of maximum grip aperture (ToMGA): time from movement initiation to the time at which the MGA occurred; (5) position of MGA (PoMGA): the distance between the object and the location where the MGA occurred; (6) normalized movement distance (NMD): the wrist trajectory distance from the start position to the touch object divided by the direct distance; and (7) grip force: mean grip force from 3.0–3.5 sec after touching the object. This period corresponded to a late holding phase, during which stable grip forces could be recorded in all participants.

For statistical analysis, we excluded the first three of 10 trials in each condition because unstable grip forces were sometimes observed. Parameters 1–6 were averaged across the seven remaining trials and three conditions in each participant. Grip force (7) was computed in each condition. Statistical analyses were performed using STATISTICA version 10.0 (StatSoft Inc., Tulsa, OK, USA), and a *p* value of <0.05 indicated statistical significance.

## 3. Results

### 3.1. Kinematics of Reach-to-Grasp Movements: Patients versus Controls

**Figure 2:**
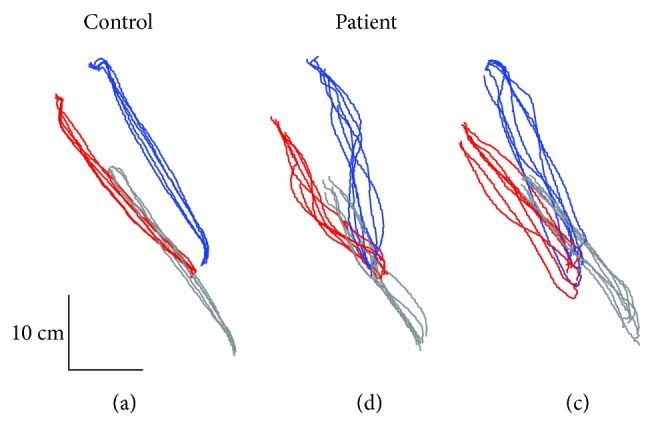
Representative examples of superimposed 2D trajectories during reach-to-grasp movements in one control subjects (a) and two patients (b–c). Red, blue, and gray lines indicate trajectories of the thumb and index finger and wrist, respectively.

All patients in the current study could complete the reach-to-grasp movement using all three materials.  Figure 2 represents the trajectories of the thumb (red), index finger (blue), and wrist (gray) during movements in one healthy control (Figure 2(a)) and two patients (Figures 2(b) and 2(c)). Qualitatively, each digit in the healthy control tended to move straight toward the target object, with the distance between the two fingertips (i.e., grip aperture) increasing from the initial position. Additionally, the healthy control showed lower intertrial variability in their movements. In contrast, the patients tended to show curved trajectories and higher variability in their movements. Additionally, the timing and size of grip aperture differed greatly among the patients. For example, some patients (Figure 2(b)) exhibited excessive grip aperture relative to object size, although the aperture started from the same initial position as in healthy controls. On the other hand, some other patients also (Figure 2(c)) tended to spread their fingers from the middle of the reaching phase, which makes PoMGA closer to the object.

**Figure 3:**
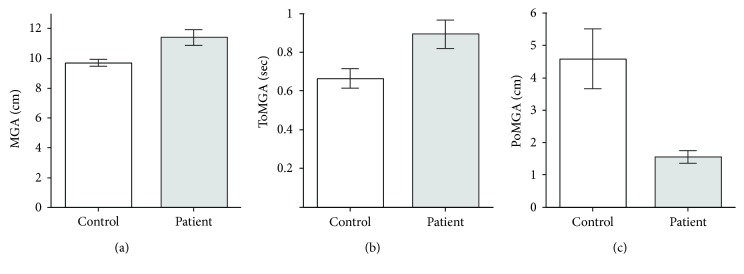
Averaged data of the control (white bars) and preoperative patients (gray bars) with regard to maximum grip aperture. (a) Maximum grip aperture (MGA). Maximum value of distance between the thumb and index finger during reach-to-grasp movements. (b) Time of maximum grip aperture (ToMGA). Time from movement initiation to time at which the MGA occurred. (c) Position of maximum grip aperture (PoMGA). The position at which the MGA occurred relative to the object.

Quantitative analysis found some significant differences between the control and patients, especially with respect to grip aperture (Figure 3). The average MGA was significantly greater in the patients (11.4 ± 2.5 cm, mean ± SD) than healthy controls (9.7 ± 1.4 cm) (*t*_32_ = 3.0, *p* < 0.01; Welch's two-sample *t-*test; Figure 3(a)). Although no difference was detected between the patients and healthy controls with respect to MT (*p* > 0.3), the patients took more time to reach the MGA (ToMGA; 0.89 ± 0.35 s versus 0.67 ± 0.28 s; patients versus controls) (*t*_42_ = 2.6, *p* < 0.05; Figure 3(b)) and consequently the location at which the MGA occurred was closer to the object (PoMGA: 1.6 ± 1.0 versus 4.6 ± 5.1 cm) (*W* = 128.0, *p* < 0.01; Wilcoxon rank-sum test; Figure 3(c)). Additionally, the NMD of patients (1.1 ± 0.1) was significantly shorter than that of controls (1.2 ± 0.2) (*t*_41_ = 3.2, *p* < 0.01; Table 2). With respect to RT, there were no significant differences between patients and controls (*p* > 0.5).

### 3.2. Grip Forces: Patients versus Controls

**Figure 4:**
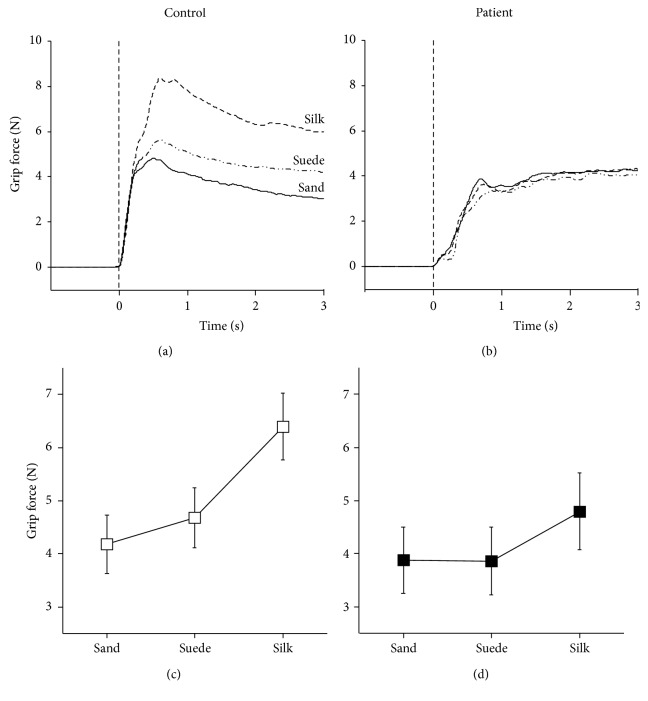
Grip force. Representative example of mean grip force profiles of one control subject (a) and one patient (b). A sold line, long dashed double-dotted line, and dashed line indicate sandpaper, suede, and silk conditions, respectively. The zero of *x*-axis (dotted vertical line) represents the starting time of lifting movement. Averaged grip forces of three conditions in the controls (c) and patients (d). Error bars denote ± 1.0SE.

Figures 4(a) and 4(b) represent the grip force profiles of three conditions in one control and one patient, respectively. The grip forces of the control increased steeply and were modulated by surface materials. The greatest force was obtained when the most slippery material was used (i.e., silk; dashed line), whereas the least force was used when the nonslip material was picked up (i.e., sandpaper; solid line). In contrast, the patient's grip force increased moderately, and material-dependent force modulations were not observed. Statistical analysis for all participants using two-way analysis of variance (ANOVA) with repeated measures in one factor (subject [2: patient and control] × material [3: silk, suede, and sandpaper]) demonstrated that the main effect of material (*F*_2, 102_ = 20.9, *p* < 0.01) and the interaction between the two (*F*_2, 102_ = 3.1, *p* < 0.05) were significant. According to post hoc multiple comparisons (Fisher's least significant difference), greater grip forces were produced under the silk condition than the sandpaper and suede conditions in controls (*p* < 0.01) and patients (*p* < 0.05) (Figures 4(c) and 4(d)). However, in comparisons between the controls and patients, only the grip force in the silk condition showed a nearly significant difference (controls versus patients: 6.4 ± 4.3 versus 4.8 ± 1.9 N; *p* = 0.07). Otherwise, there were no differences between the two groups (*p* > 0.3).

### 3.3. Postoperative Changes

Immediately after surgery, 15 of 23 patients who underwent surgery repeated the same prehension task. Hence, we compared the data between the pre- and postoperative states. In the reach-to-grasp movement, RT (pre versus post; 0.52 ± 0.25 versus 0.46 ± 0.22 s: *t*_14_ = 2.05, *p* = 0.06, paired *t*-test) and MT (1.15 ± 0.55 versus 0.94 ± 0.32 s: *t*_14_ = 1.81, *p* = 0.09) tended to decrease after surgery (Table 2), although these differences did not reach statistical significance (*p* = 0.05). For the remaining parameters (i.e., MGA, ToMGA, PoMGA, and NMD), no significant differences were detected (*p* > 0.2). With respect to grip force, two-way ANOVA (period [2: pre- and postoperation] × material [3: silk, suede, and sandpaper]) showed that the main effect of period (*p* > 0.2) and the interaction between the two (*p* > 0.8) were not significant. However, the main effect of material was statistically significant (*F*_2, 28_ = 8.4, *p* < 0.01). Post hoc analysis using Tukey's honest significant difference test found that grip force in the silk condition (4.7 ± 2.5 N) was significantly greater than that in the sandpaper condition (3.3 ± 1.7 N; *p* < 0.01).

### 3.4. JOA Scores for the Upper Extremity

The JOA score for the upper extremity showed that the preoperative patients had obvious damage in two domains: motor (1.9 ± 1.1, normal = 4: *t*_22_ = 8.9, *p* < 0.01, one sample *t*-test) and sensory (1.1 ± 0.5, normal = 2: *t*_22_ = 8.6, *p* < 0.01) scores (Table 2). The total score of motor and sensory domains was 3.0 ± 1.3 (normal = 6; *t*_22_ = 10.9, *p* < 0.01). For the patients who underwent surgery (*n* = 15), we examined whether postoperative recovery could be observed using a paired *t*-test or Wilcoxon signed-rank test. As a result, significant improvements after surgery were identified with respect to total score (pre- versus postoperative: 3.2 ± 1.6 versus 3.8 ± 1.3; *t*_14_ = 2.78, *p* < 0.05). A similar tendency was also observed for sensory function (*t*_14_ = 1.95, *p* = 0.07) but not for motor function (*p* > 0.1; Wilcoxon signed-rank test).

### 3.5. Regression and Correlation Analyses

To verify the clinical validity of the quantitative assessment system for hand dexterity impairment applied in this study, we performed regression analyses between parameters obtained from the test and the JOA score. Here, we used the combined data (*n* = 38) of the pre- and postoperative patients.  Table 3 shows the correlation coefficients between motor and sensory scores of the JOA scale and the parameters of the task. The motor and sensory scores of the JOA scale did not correlate significantly.

Some parameters correlated with JOA scores. For example, the motor score significantly correlated with MT (*r* = −0.63; *p* < 0.01) and RT (*r* = −0.47; *p* < 0.01). Although the motor score evaluates dexterous finger movements (Table 1), it did not correlate with grip forces irrespective of the surface material of the object. In contrast, the sensory score correlated significantly with a different parameter: grip force of the suede condition (*r* = −0.33; *p* < 0.05). Interestingly, the correlation coefficient showed a negative value; patients with good sensory function exerted weaker grip force in the condition.

**Figure 5:**
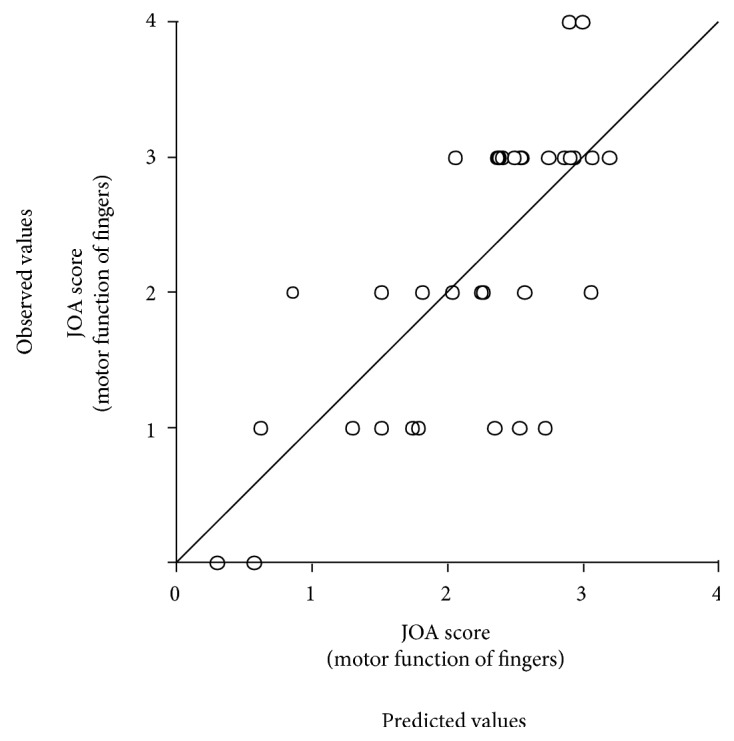
A scatter plot regarding the JOA score (motor function of fingers: normal = 4) between predicted values according to a multiple regression analysis (abscissa) and the observed values (ordinate). A diagonal line means an exact match between the two values.

We further examined whether parameters obtained from the current task (explanatory variables) could explain the motor score assessed from the JOA score (object variable) using multiple regression analysis and selected explanatory variables to yield an appropriate regression model using the forward-stepwise method. As a result, a significant regression model was found (*F*_5, 30_ = 8.09, *p* < 0.01, adjusted *r* ^2^ = 0.50).  Figure 5 shows a scatter plot between the motor scores predicted from the model (abscissa) and the observed scores (ordinate). The significant predictors were MT (*β* = −0.91, *p* < 0.01), ToMGA (*β* = 0.47, *p* < 0.05), and grip forces in the suede (*β* = −0.55, *p* < 0.05) and silk conditions (*β* = 0.48, *p* < 0.05).

Similar analyses were performed for sensory and total scores. The sensory score could not be explained by the current variables (*F*_2, 33_ = 2.67, *p* = 0.08, adjusted *r* ^2^ = 0.09). The total JOA score showed an intermediate value between motor and sensory scores (*F*_5, 30_ = 4.57, *p* < 0.01, adjusted *r* ^2^ = 0.34). Significant predictors for the latter were MT (*β* = −0.71, *p* < 0.05) and grip force in the suede condition (*β* = −0.58, *p* < 0.05).

## 4. Discussion

In this study, we used a natural prehension movement task to quantitatively examined hand dexterity impairment in patients with CM. Previously, this task has been used in studies of monkeys to verify dexterous finger movement ability after damage to the neuronal system [13, 14]. For this reason, we believed that it would be useful for assessing hand dexterity in patients with CM and aimed to test this hypothesis.

In the present study, preoperative patients had decreased performance compared to controls, mainly with respect to grip aperture control and grip force modulation. Immediately after surgery, the patients showed improvements in JOA score and shortening of RT and MT compared to controls. Multiple regression analysis demonstrated that several parameters from the prehension task could explain the dysfunctions of finger movements in daily life according to the JOA score. These results suggest that analysis of this natural prehension task shows promise for objectively evaluating the current severity of CM with respect to hand dexterity impairments.

Traditionally, the reach-to-grasp movement is thought to consist of two components: transport or reaching (i.e., the hand is moved toward the object) and grasping (i.e., hands or fingers are preshaped in anticipation of contact with the object) [15]. Previous studies in healthy humans have reported that the MGA occurs at approximately 60–70% of the reaching duration [16, 17], suggesting a tight coupling between the two components. The current results in age-matched controls were consistent with this principle (66%, 0.67 s/1.01 s, ToMGA/MT). However, in the patients, the MGA occurred much later (80%, 0.90 s/1.12 s) and immediately before contact with the object (i.e., PoMGA). Moreover, the patients showed evidence of excessive MGA compared to the controls. We assume that changes in grip aperture control were caused by pyramidal tract damage in the spinal cord [13]. However, these changes may also reflect decreased somatosensory information associated with CM (sensory score of JOA: 1.11 ± 0.45; normal = 2). Gentilucci et al. [18] examined the role of tactile information from the hand during reach-to-grasp movements by providing local anesthesia to the participants' fingertips. The results showed that blocking tactile afferents mainly influenced the kinematics of the finger-opening phase: the duration of this phase was extended and MGA increased. The results in patients in this study are consistent with these findings and might provide evidence for the importance of somatosensory inputs from the hand in grip aperture control.

When picking up a small object up using the index finger and thumb, people can modulate their grip force adequately based on the friction between the skin of the finger and the object (i.e., the more slippery the object, the greater the grip force) [12]. However, the force modulation ability of CM patients was decreased in this study. In particular, patients were unable to exert higher grip force when attempting to pick up the most slippery object (i.e., silk) when compared to controls. There are at least two reasons for impaired grip force modulation in these patients. One is that the patients' finger muscle strength was decreased due to CM so that the patients could not produce a strong grip force despite being able to complete the task. It is known that patients with CM show lower scores (<20 times) on the 10 sec grip-and-release test [10], which is correlated with gripping power to some extent [19]. Hence, in this study, we infer that patients' maximum grip forces would be weakened. The other reason is that patients' failure to adapt to different surficial materials (i.e., frictions) was caused by sensory dysfunction in the hand, as mentioned above. A human study involving the administration of local anesthesia to the index finger and thumb demonstrated that the adaptation to friction between the skin and the object was strongly dependent on cutaneous afferent input [12].

In this study, in contrast to the silk condition, the grip forces under the suede and sandpaper conditions in patients were not different from those of controls. Because the patients could exert stronger forces in the sandpaper condition, the forces of these two conditions could be less influenced by muscle strength. Indeed, the forces correlated moderately with the sensory JOA score. If patients have a good sensory function, they use weaker grip forces for the materials, as observed in healthy controls [12].

Postoperatively, JOA score was significantly improved, although it was still far from the normal value. In the prehension task, postoperative changes were confirmed only in the reaching component (i.e., shortening of the RT and MT) but not in the grasping and lifting components. The difference between the reaching and grasping (or lifting) components could be partially explained by direct and indirect motor pathways from the motor cortex to spinal motoneurons during control of the upper extremity. Animal studies in cats and monkeys have demonstrated that reaching movements are less influenced than finger manipulations after spinal pyramidotomy (i.e., surgical severance of the direct pathway [i.e., pyramidal tract] by creating a partial lesion of the lateral funiculus) at the C5 level [13, 14]. This effect would be caused by an indirect pathway (i.e., interneuronal systems), including propriospinal neurons at the C3–C4 level, which mediate corticomotoneuronal inputs to the proximal arm muscles predominantly [20]. The existence of the C3–C4 propriospinal neuron system in humans has also been suggested from electrophysiological findings [21, 22]. Accordingly, the current results might reflect different time courses of recovery processes between the direct and indirect pathways after surgery. Similar dissociation in movement impairments and their recovery was also observed in patients with CM [11]. To corroborate this hypothesis, we need to perform further follow-up investigations of postoperative changes using this prehension task.

While the grasping and lifting components are unable to detect clear recovery immediately after surgery, our multiple regression analysis showed that the parameters in the prehension task could explain the current motor dysfunction of fingers in activities of daily living (e.g., fastening buttons) according to the JOA score (motor score) (adjusted *r* ^2^ = 0.50; *p* < 0.01). The selected explanatory variables included all movement components (reaching [MT], grasping [ToMGA], and lifting [grip forces of the suede and silk conditions]). At first sight, it is unusual that the reaching component (MT) is included in the variables because the reaching and grasping components are independent [15]. However, in a study of monkeys involving pyramidotomy, it was shown that the animal could pinch food pellets with the index finger and thumb after a recovery period [13, 14]. The authors suggested that this recovery could be induced by interneuronal systems, which mediate corticomotoneuronal inputs to the proximal arm muscles in the normal condition [20]. Thus, it is possible that the interneuronal systems could contribute to the recovery of dexterous hand movements even in patients with CM. The contribution of interneuronal systems was also suggested in a previous study with CM patients [11]. Otherwise, the ToMGA and grip forces of the suede and silk conditions indicate the functioning of the pyramidal tract, sensory functions, and muscle strength of the hand muscles. Thus, dexterous hand movements are supported by different neuronal systems.

There are several limitations to this study should be discussed. First, the CM patients in the current study could complete the task. Thus, the current method cannot be applied to patients with severe impairments. Second, we did not follow the recovery of patients over a long-term period. To evaluate recovery in the long term, future studies will be needed. Third, we fixed the order of the surface materials to make the task easier for patients. Finally, we analyzed data from patients with compression at different spinal levels. In the future, we need to collect detailed data to the clarify effects of compression level on the findings of the present study.

## 5. Conclusions

The results of this study suggest that prehension movement analysis could be efficient and valid for the objective evaluation of current impairment of hand dexterity in patients with CM.

## Figures and Tables

**Table 1:** Japanese Orthopedic Association score for cervical compressive myelopathy.

Dysfunction score
A. Motor function
I. Fingers
0 = unable to feed oneself with any tableware, including chopsticks, a spoon, or fork, and/or unable to fasten buttons of any size
1 = can manage to feed oneself with a spoon and/or a fork but not with chopsticks
2 = either chopstick feeding or writing is possible but not practical, and/or large buttons can be fastened
3 = either chopstick feeding or writing is clumsy but practical, and/or cuff buttons can be fastened
4 = normal
II. Shoulder and elbow (evaluated by MMT score of the deltoid or biceps muscles, whichever is weaker)
−2 = MMT ≤ 2
−1 = MMT 3
−0.5 = MMT 4
0 = MMT 5
III. Lower extremity
0 = unable to stand up and walk by any means
0.5 = able to stand up but unable to walk
1 = unable to walk without a cane or other support on a level
1.5 = able to walk without a support but with a clumsy gait
2 = walks independently on a level but needs support on stairs
2.5 = walks independently when going upstairs but needs support when going downstairs
3 = capable of fast walking but clumsily
4 = normal
B. Sensory function
I. Upper extremity
0 = complete loss of touch and pain sensation
0.5 = 50% or below of normal sensation and/or severe pain or numbness
1 = over 60% of normal sensation and/or moderate pain or numbness
1.5 = subjective numbness of a slight degree without any objective sensory deficit
2 = normal
II. Lower extremity (same as I)
III. Trunk (same as I)
C. Bladder function
0 = urinary retention and/or incontinence
1 = sense of retention and/or dribbling and/or thin stream and/or incomplete continence
2 = urinary retardation and/or pollakiuria
3 = normal

MMT: manual muscle testing.

**Table 2:** Summarized data from prehension movement analysis and JOA score.

Parameter	Control	Patient	
		Preop	Postop
Number male/female	30 12/18	23 15/8	15 12/3
Age	63.4 ± 17.2	65.0 ± 14.8	63.9 ± 15.8
Reach-to-grasp movement			
Reaction time [RT] (sec)	0.53 ± 0.19	0.50 ± 0.24	0.46 ± 0.22 (0.52 ± 0.25)
Movement time [MT] (sec)	1.01 ± 0.37	1.12 ± 0.43	0.94 ± 0.32 (1.15 ± 0.55)
Maximum grip aperture [MGA] (cm)	9.71 ± 1.35	11.4 ± 2.46^∗∗^	11.7 ± 2.62 (11.5 ± 2.53)
Time of maximum grip aperture [ToMGA] (ms)	0.67 ± 0.28	0.89 ± 0.35^∗^	0.78 ± 0.33 (0.91 ± 0.44)
Position of maximum grip aperture [PoMGA] (cm)	4.58 ± 5.09	1.55 ± 0.96^§§^	1.27 ± 0.67 (1.43 ± 0.70)
Normalized movement distance [NMD]	1.22 ± 0.18	1.11 ± 0.07^∗∗^	1.08 ± 0.05 (1.10 ± 0.08)
Grip force (N)			
Sandpaper	4.18 ± 3.56	3.88 ± 2.08	3.88 ± 2.08
Suede	4.68 ± 3.81	3.86 ± 1.68	3.73 ± 1.84 (4.24 ± 1.82)
Silk	6.39 ± 4.27	4.79 ± 1.94	4.34 ± 2.84 (5.06 ± 2.14)
JOA score			
Motor function of fingers	—	1.91 ± 1.12	2.40 ± 1.12 (2.13 ± 1.25)
Sensory function of the upper extremity	—	1.11 ± 0.50	1.37 ± 0.52 (1.10 ± 0.47)
Total (motor + sensory)	—	3.02 ± 1.31	3.77 ± 1.28^†^ (3.23 ± 1.55)

Values are expressed as mean ± SD. JOA test was not performed for normal controls (−). Values in parentheses indicate preoperative values of 15 patients.^∗^Statistically significant difference from control (*p* < 0.05, Welch two sample *t*-test). ^∗∗^ Statistically significant difference from control (*p* < 0.01, Welch two sample *t*-test). ^§§^Statistically significant difference from control (*p* < .01, Wilcoxon rank-sum test). ^†^Statistically significant different from preoperation (*p* < .05, paired sample *t*-test).

**Table 3:** Correlation coefficients between all parameters using combined data from pre-and postoperative patients (*n* = 38).

Parameter	2	3	4	5	6	7	8	9	10	11
1. JOA (motor)	.31	−.47^∗∗^	−.63^∗∗^	−.28	−.06	−.21	−.32	−.11	−.15	.09
2. JOA (sensory)		.05	−.03	−.11	−.05	.16	−.15	−.26	−.33^∗^	−.24
3. RT			.59^∗∗^	.42^∗∗^	.27	.01	.48^∗∗^	.02	−.12	−.27
4. MT				.71^∗∗^	−.06	.20	.41^∗^	.02	.07	−.06
5. MGA					.13	.05	.41^∗^	−.02	−.12	−.14
6. ToMGA						−.08	.33	.10	.10	−.06
7. PoMGA							.27	.10	.03	−.04
8. NMD								.27	.18	.17
9. Grip force (sand)									.68^∗∗^	.44^∗∗^
10. Grip force (suede)										.80^∗∗^
11. Grip force (silk)										

^∗^ *p* < 0.05, ^∗∗^ *p* < 0.01. JOA: Japanese Orthopedic Association score; RT: reaction time; MT: movement time; MGA: maximum grip aperture; ToMGA: time of maximum grip aperture; PoMGA: position of maximum grip aperture; NMD: normalized movement distance.

## Data Availability

The datasets used to support the findings of this study are available from the corresponding author upon request.

## Supplementary Materials

Supplementary Materials Table S1: *W* values and *p* values within parenthesis obtained from the comparison between men and women using Wilcoxon rank-sum test. Table S2: *D* values and significances from Kolmogorov-Smirnov goodness-of-fit test.
