# Supplementary material for: Hand Dexterity Impairment in Patients with Cervical Myelopathy: A New Quantitative Assessment Using a Natural Prehension Movement
Source: Behav Neurol. 2018 Jul 4;2018:5138234. doi: 10.1155/2018/5138234 (PMC6057419; doi:10.1155/2018/5138234)
Supplement: Supplementary Materials — Table S1: W values and p values within parenthesis obtained from the comparison between men and women using Wilcoxon rank-sum test. Table S2: D values and significances from Kolmogorov-Smirnov goodness-of-fit test. [file 5138234.f1.pdf]

Table S1: W-values and p-values within parenthesis from Wilcoxon rank-sum test

Parameter	Control	Patient
		Pre-op
Number	30	23
Male / Female	12/18	15/8
Reach-to-grasp movement		
Reaction time [RT] (sec)	97.0 (0.66)	50.0 (0.54)
Movement time [MT] (sec)	108.0 (1.0)	56.0 (0.82)
Maximum grip aperture [MGA] (cm)	105.0 (0.92)	40.0 (0.21)
Time of maximum grip aperture [ToMGA] (ms)	98.0 (0.69)	38.0 (0.23)
Position of maximum grip aperture [PoMGA] (cm)	77.0 (0.20)	56.0 (0.82)
Normalized movement distance [NMD]	81.0 (0.26)	50.0 (0.54)
Grip force (N)		
Sandpaper	64.0 (0.07)	47.0 (0.42)
Suede	62.0 (0.054)	53.0 (0.67)
Silk	66.0 (0.08)	54.0 (0.72)
JOA score		
Motor function of fingers	–	53.0 (0.67)
Sensory function of upper extremity	–	43.5 (0.30)
Total (motor + sensory)	–	47.5 (0.44)

Table S2: D-values and significances from Kolmogorov-Smirnov goodness-of-fit test

		Patient	
Parameter	Control	Pre-op	Post-op
Number	30	23	15
Male / Female	12/18	15/8	12/3
Reach-to-grasp movement			
Reaction time [RT] (sec)	0.16	0.25	0.19 (0.20)
Movement time [MT] (sec)	0.19	0.12	0.16 (0.11)
Maximum grip aperture [MGA] (cm)	0.16	0.20	0.26 (0.26)
Time of maximum grip aperture [ToMGA] (ms)	0.10	0.20	0.12 (0.21)
Position of maximum grip aperture [PoMGA] (cm)	0.25 *	0.15	0.16 (0.13)
Normalized movement distance [NMD]	0.22	0.14	0.17 (0.20)
Grip force (N)			
Sandpaper	0.17	0.12	0.30 (0.16)
Suede	0.18	0.11	0.31 (0.13)
Silk	0.17	0.16	0.27 (0.22)
JOA score			
Motor function of fingers	–	0.18	0.37 * (0.29)
Sensory function of upper extremity	–	0.24	0.20 (0.32)
Total (motor + sensory)	–	0.12	0.17 (0.22)

* $p < 0.05$

Values in parentheses indicate preoeprative values of 15 patients.
